# Distinct Neuropsychological Correlates of Cognitive, Behavioral, and Affective Apathy Sub-Domains in Acquired Brain Injury

**DOI:** 10.3389/fneur.2014.00073

**Published:** 2014-05-19

**Authors:** Progress Njomboro, Shoumitro Deb

**Affiliations:** ^1^Department of Psychology, University of Cape Town, Cape Town, South Africa; ^2^Division of Brain Sciences, Department of Medicine, Imperial College London, London, UK

**Keywords:** apathy, affective, cognitive, behavioral, depression

## Abstract

Apathy has a high prevalence and a significant contribution to treatment and rehabilitation outcomes in acquired brain damage. Research on the disorder’s neuropsychological correlates has produced mixed results. While the mixed picture may be due to the use of varied assessment tools on different patient populations, it is also the case that most studies treat apathy as a unitary syndrome. This is despite the evidence that apathy is a multifaceted and multidimensional syndrome. This study investigates the neuropsychological correlates of apathy in 49 patients with acquired brain damage. It further fractionates apathy symptoms into affective, cognitive, and behavioral sub-domains and investigates their individual relations with standard measures of affective, cognitive, and behavioral functioning. Global apathy scores were not related to any of these measures. Affective apathy was associated with emotion perception deficits, and cognitive apathy was associated with executive deficits on the Brixton test. These results demonstrate that treating apathy as a single entity may hide important correlates to apathy symptoms that become visible when the disorder is fractionated into its sub-domains. The study highlights the research and clinical importance of treating apathy as a multidimensional syndrome.

## Introduction

Since Marin’s seminal work on apathy in the early 1990s, there has been a tremendous growth in the amount of research on the disorder and a general appreciation of its clinical relevance. It is now widely agreed that apathy constitutes a quantitative reduction in self-generated voluntary and purposeful acts, and that this reduction in activity reflects dysfunctions in affective, cognitive, and behavioral processes relating to the planning, execution, and control of goal directed behavior (1–5). Prevalence studies show that apathy is the most common long-term clinical feature following brain injury (3, 6). A meta-analytical study covering 19 studies with a total of 2221 patients by Caeiro et al. (7) found that the frequency of apathy across studies ranged between 15.2 and 71.1% [see also Ref. (8, 9) for similar findings]. Patients with apathy present with more negative rehabilitation outcomes in terms of poor recovery (6, 10, 11), problems in daily functioning (12), lack of post-injury social reintegration (13), loss of social autonomy (14), financial and vocational loss (8), significant neuropsychological dysfunction (2, 7, 12, 15–18), and caregiver distress (19). Yet, apathy is still a neglected neuropsychiatric syndrome in clinical practice, with no known standard treatment approaches and remains largely excluded from major psychiatric disease classification systems.

Apathy’s position in relation to other neuropsychiatric conditions is also less clear, particularly its relationship to negative symptoms, dysexecutive syndrome, or affective illness (20–22). Studies investigating the neural substrates of apathy implicate a diverse range of cortical and sub-cortical brain areas (23–25). This lack of clarity probably reflects the lack of consensus on the definition of apathy, or the use of different clinical measures and neuropsychological tests and/or different neuropsychiatric patients across studies. It is, however, important to also note that the majority of studies treat apathy as a unitary disorder, while it is actually a multifaceted syndrome with distinct affective, cognitive, and behavioral sub-domains (2, 5, 20). The prominence of any one of these sub-domains on the clinical profile depends on the specific underlying neural involvement in neuropsychiatric illness (23–26). For instance, Sultzer et al. (25) found that: (1) affective apathy symptoms are associated with low metabolism in left medial temporal, right anterior temporal, and left inferior frontal cortex, (2) cognitive apathy symptoms are associated with low metabolic activity in bilateral medial thalamus, bilateral anterior cingulate, and left insula, and (3) behavioral apathy symptoms are associated with low activity in bilateral insula. Chow et al. (24) also point out that affective symptoms are linked to damage in emotion related sub-cortical circuits involving the limbic structures, while cognitive symptoms are associated with frontal lobe dysfunction [see also Ref. (23, 26, 27)].

This study investigates the correlates of apathy symptoms while controlling for the contribution of individual apathy sub-domains to neurocognitive functioning. Isolating these sub-domains and investigating their individual relations to neuropsychological performance has both theoretical validity and empirical utility. This approach takes into account the multifaceted nature of apathy symptoms and acknowledges the importance of treating these domains separately when investigating the neuropsychological profiles that may accompany them. Furthermore, imaging studies with neurologically intact participants indicate that the processes that are disabled in apathy, such as those involved in self-initiation, emotional expression and feeling, and volition, are neurally distinct (24, 25). Fractionating apathy symptoms into affective, cognitive, and behavioral sub-domains may help us better investigate these associated neurobehavioral correlates. Previous studies have also tended to use homogeneous patient samples, making it difficult to isolate apathy correlates from those related to the specific disease process. This study includes a heterogonous sample of brain damaged patients in order to dilute the influence of disease-specific effects and strengthen the relations between apathy symptoms and the study variables.

The main aim of this study is to identify the neuropsychological correlates of the affective, cognitive, and behavioral sub-domains of apathy. The speculation is that each of these three sub-domains has distinct correlates. Specifically, three hypotheses are made. Hypothesis (1): affective apathy symptoms predict poor outcomes on affective measures in terms of more depressive symptoms on the Beck depression inventory (BDI) and also predict poor perception of basic emotions on the Emotion Hexagon test; Hypothesis (2): cognitive apathy symptoms predict poor performance on a cognitive test of executive function (the Brixton test); and Hypothesis (3): behavioral apathy symptoms predict poor goal directed behavior on the Iowa gambling task (IGT).

## Materials and Methods

### Participants

Participants for this study were recruited from clinics and rehabilitation centers within the United Kingdom’s city of Birmingham. In total, 49 patients with acquired brain damage were recruited. All the patients were at least 6 months post-injury and were screened through the Birmingham Cognitive Screen (28) to establish suitability for participation in the study (see Table 1 for the cause of injury and patients’ demographic characteristics).

**Table 1:** **Patients’ demographic characteristics and cause of brain injury**.

Etiology	*N*	Sex	Age
		M = male; F = female	Mean and SD
Cerebrovascular accident (CVA)	24	M = 15; F = 9	54.44 (12.29)
Head injury	14	M = 11; F = 3	48.25 (15.27)
Anoxia	5	M = 5; F = 0	49.80 (11.05)
Herpes simplex encephalitis (HSE)	6	M = 5; F = 1	42.50 (8.60)
Total	49	M = 36; F = 13	48.75 (11.80)

Imaging results and lesion data were obtained from patient files and were available in 46 patients (see Table 2). All participants gave informed written consent, and the ethics approval for the study was granted by the Birmingham and Solihull Research Ethics Committee.

**Table 2:** **Lesion location for study participants**.

Lesion location	Number of patients
Left parietal	5
Right parietal	4
Bilateral parietal	1
Left fronto-temporal	7
Right fronto-temporal	7
Bilateral fronto-temporal	22
Total (*N*)	46

## Measures

### Assessment of apathy

The informant rated Apathy Evaluation Scale (AES-I) (20) was used to assess apathy symptoms. The AES is the most widely used scale for evaluating apathy symptoms and has demonstrated good psychometric properties (21). The 18 items on the scale assess behavioral apathy symptoms (e.g., *He/she spends time doing things that interest her/him*), emotional apathy symptoms (e.g., *When something good happens, he/she gets excited*), and cognitive apathy symptoms (e.g., *S/he is interested in things*). Each item is rated on a scale of 1 (*Not at all*) to 4 (*A lot*).

### Assessment of affective functioning

#### Depression

The level and presence of depressive symptoms was evaluated using the BDI II (29).

#### Emotion perception

The Emotion Hexagon test from the facial expressions of emotions stimuli and tests (FEEST) CD-ROM (30) was used to assess the recognition of facial expressions of emotion. The test assesses recognition of the six basic emotional categories of anger, disgust, fear, happiness, sadness, and surprise. The pictures of faces on the test are computer-manipulated and morphed such that each emotion is presented in graded levels of difficulty making the emotions relatively difficult to recognize. For that reason, the Hexagon test is a more valid measure of emotion perception because in real-life emotions are seldom expressed as a single pure emotion, but as complex combinations of mixed emotions. The test is made up of a total of 120 trials split into four blocks. Participants respond by using a mouse to click on one of the six emotion words (i.e., Anger, Disgust, Happiness, Sadness, Surprise, and Fear) presented at the bottom of a computer screen together with each emotional face; see Young et al. (30) for a full description of the test.

### Assessment of cognitive functioning

#### The Brixton test

The Brixton Spatial Anticipation test (31) provided a measure of executive cognitive control. This test was chosen because of its limited semantic loading, and its sensitivity to deficits in a number of executive processes, including set shifting and rule or feedback use (32). The test consists of a series of pages in a booklet. Each page has the same basic design on it made up of a set of 10 circles in 2 rows. One of the circles among these 10 circles is colored blue, and the participants’ task is to infer patterns in the sequence of positions of the blue circle and predict its position on subsequent pages.

### Assessment of behavioral functioning

#### Iowa gambling task

The IGT assesses motivational decision making and performance on this task is thought to analog real-life goal directed behavior (33). The task requires participants to choose cards from four decks labeled A–D containing 40 cards each. Participants win and lose money on all the decks, but decks A and B give higher wins but even higher losses while decks C and D give relatively lower wins and similarly lower losses. Picking more cards from decks A and B gives a net loss while picking from decks C and D results in a net gain. Neurologically intact participants tend to avoid decks A and B and pick more from decks C and D as the game progresses (33). Successful performance on the task is reflected by the number of cards picked from decks C and D. For each participant, the IGT score was the number of cards selected from these two decks.

## Data Analysis

Initially, a Pearson correlation analysis was performed to investigate the relationship between global apathy (total AES-I score) and performance on the tests. Further multiple regression analyses were performed to investigate the influence of each of the three sub-domains of apathy (i.e., affective apathy, cognitive apathy, and behavioral apathy) on test performance. The total sub-domain score was obtained by summing up scores on all the AES-I items under that domain. A series of multiple linear regression analyses were then performed with the sub-domain scores treated as predictors of performance on the individual tests. The data analysis was done using SPSS Version 21 software (IBM, Armonk, NY, USA). All the data were tested for violations of the assumptions of normality, linearity, and homoscedasticity.

## Results

### Neuropsychological correlates of global apathy

Global apathy was not significantly related to any of the test measures. These results are shown in the correlation matrix in Table 3 below.

**Table 3:** **Correlations between global apathy and tests scores**.

		Hexagon	IGT	Brixton	AES-I
IGT	Coefficient	0.06			
	*Sig*.	*0.63*			
Brixton	Coefficient	0.08	0.17		
	*Sig*.	*0.56*	*0.37*		
AES-I	Coefficient	0.07	0.30	0.17	
	*Sig*.	*0.67*	*0.11*	*0.29*	
BDI	Coefficient	0.18	0.01	0.10	0.06
	*Sig*.	*0.23*	*0.99*	*0.50*	*0.70*

Hierarchical regression analyses were then performed to examine the individual contribution of affective, cognitive, and behavioral apathy sub-domains to performance on the individual neuropsychological tests. The results for each sub-domain are presented in the following sections below.

### Affective apathy and depressive symptoms

Hypothesis 1 suggested that affective apathy symptoms would be positively correlated to depressive symptoms. For this reason, affective apathy scores were entered in the first step of the regression model with depressive symptoms scores, and then cognitive and behavioral apathy scores were entered in the second step. Affective apathy symptoms were not a significant predictor of depressive symptoms in the first step of the model. Controlling for the influence of cognitive and behavioral apathy symptoms in the second step of the regression model also produces non-significant effects. These results are shown in Table 4.

**Table 4:** **The relationship between apathy domains and depressive symptoms**.

	*B*	SE *B*	β	*p*
Step 1
Constant	14.48	4.70		
Affective apathy	−0.04	0.94	−0.01	0.99
Step 2
Constant	11.39	6.99		
Affective apathy	0.58	1.45	0.09	0.69
Cognitive apathy	−0.41	0.46	−0.25	0.38
Behavioral apathy	0.70	0.79	0.19	0.38

*R^2^ = 0 for step 1; Δ*R*^2^ = 0.02 for step 2 (*p*s > 0.05)*.

### Affective apathy and emotion perception

It had also been suggested in Hypothesis 1 that affective apathy symptoms would be associated with poor emotion recognition on the Emotion Hexagon test. Affective apathy scores were entered in the first step of the model together with the Hexagon test scores, followed by scores on cognitive and behavioral apathy symptoms in the second step. The influence of affective apathy symptoms on emotion perception was not significant in the first step of the model but was significant in step 2 after factoring in cognitive and behavioral symptoms. These results are shown in Table 5.

**Table 5:** **Apathy and emotion recognition on the Emotion Hexagon test**.

	*B*	SE *B*	β	*p*
Step 1
Constant	92.33	8.67	
Affective apathy	−2.42	1.73	−0.20	0.17
Step 2
Constant	80.00	12.55	
Affective apathy	−5.85	2.60	−0.48	0.03*
Cognitive apathy	0.92	0.83	0.29	0.27
Behavioral apathy	0.85	1.41	0.12	0.55

*R^2^ = 0.04 for step 1; Δ*R*^2^ = 0.07 for step 2 (*p*s > 0.05)*.

*p < 0.05.

### Cognitive apathy and executive function

In Hypothesis 2, it has been predicted that higher cognitive apathy symptoms would be associated with poorer executive function performance on the Brixton test. Cognitive apathy scores were entered in the first step of the regression model together with Brixton scores, followed by affective and behavioral apathy scores in the second step. The contribution of cognitive apathy in the first step of the regression model neared significance (*p* = 0.06), suggesting that cognitive apathy symptoms are related to poor Brixton test performance. There were no significant predictors of executive function impairment on the Brixton test in the second step of the regression analysis (see Table 6).

**Table 6:** **Apathy domains and the Brixton test**.

	*B*	SE *B*	β	*p*
Step 1
Constant	18.89	3.51		
Cognitive apathy	0.31	0.17	0.26	0.06
Step 2
Constant	21.03	4.85		
Cognitive apathy	0.35	0.32	0.29	0.28
Affective apathy	0.50	1.00	0.11	0.62
Behavioral apathy	−0.45	0.54	−0.17	0.42

*R^2^ = 0.07 for step 1; Δ*R*^2^ = 0.02 for step 2 (*p*s = 0.06); *p* = 0.06*.

### Behavioral apathy and motivational decision making

Hypothesis 3 speculated that behavioral apathy scores would predict poor performance on the IGT, in line with the conceptualization of this task as an analog to real-life goal directed behavior. Behavioral apathy scores were entered in the first step of the regression analysis together with IGT scores, and then followed by cognitive and affective apathy scores in the second step. None of the three apathy sub-domains was a significant predictor of performance on the IGT. The results from the regression model are shown in Table 7.

**Table 7:** **Apathy domains and the Iowa gambling task**.

	*B*	SE *B*	β	*p*
Step 1
Constant	49.38	14.27		
Behavioral apathy	−0.37	1.14	−0.06	0.75
Step 2
Constant	54.36	13.03		
Behavioral apathy	2.23	1.46	0.38	0.14
Cognitive apathy	−0.90	0.87	−0.34	0.31
Affective apathy	−3.97	2.70	−0.39	0.15

*R^2^ = 0.01 for step 1; Δ*R*^2^ = 0.27 for step 2 (*p*s > 0.05)*.

## Discussion

This study examined the predictive value of apathy symptoms on performance on a set of affective, cognitive, and behavioral neuropsychological tests. We found that cognitive apathy symptoms were associated with more errors on the Brixton test. This result was of borderline significance (*p* = 0.06). Affective apathy symptoms were not associated with poor emotion recognition on the Emotion Hexagon test, although adding cognitive and behavioral symptoms in the second part of the regression model showed significant interaction effects of these apathy sub-domains on emotion recognition. Affective apathy symptoms were not significant predictors of depressive symptoms on the BDI. Also, behavioral apathy symptoms were not significant predictors of performance on the IGT as had been expected. These results, particularly on the relation between cognitive apathy symptoms and executive function, and also on the relationship between the three apathy sub-domains and emotion recognition are interesting considering that our initial correlation analyses investigating these relationships using global apathy scores yielded non-significant relations. Overall, these results suggest a differential influence of the three dimensions of apathy symptoms on neuropsychological performance. The following sections discuss these results in more detail.

### Correlates of affective apathy

#### Depression

Depressive symptoms were not significantly correlated to either global apathy or to the affective sub-symptoms of apathy. Other studies have reported similar findings [e.g., Ref. (34–37)]. These results support the view that apathy is a distinct neuropsychiatric syndrome separable from depression (38). There is also evidence that apathy is not a clinical criterion of depression, even though some depression scales treat it as one (21). Taken in this context, the lack of a significant association between affective apathy symptoms and depressive symptoms is unremarkable.

#### Emotion recognition

The unique contribution of affective apathy symptoms to emotion recognition deficits was not significant [although adding cognitive and behavioral apathy symptoms to the regression model produced significant effects (*p* = 0.03)]. Other studies with different clinical samples have reported poor emotion recognition in patients with apathy. For example, Martínez-Corral et al. (39) found that Parkinson’s disease patients with apathy exhibited selective deficits in facial emotion recognition, with more specific deficits in the recognition of fear, anger, and sadness, while non-apathetic patients recognized facial emotions as accurately as healthy controls [however, see Ref. (40) for a different set of results]. Drapier et al. (41) suggest that apathy and emotion recognition share the same functional circuit involving the sub-thalamic nucleus (STN).

### Correlates of cognitive apathy

Higher cognitive apathy symptoms predicted more executive function deficits on the Brixton test. Numerous studies have also linked apathy to executive dysfunction (16, 42–44). Levy and Dubois (17) have termed the cognitive sub-syndrome of apathy “cognitive inertia,” in which there is a reduction in goal directed behavior due to impairments in the higher order functions responsible for the cognitive control of activity. It might be the case that an apathetic state with predominantly more cognitive apathy symptoms would manifest as a dysexecutive syndrome.

### Correlates of behavioral apathy

Behavioral apathy scores were not significant predictors of IGT performance in this study. Our hypothesis had been informed by studies suggesting that reward insensitivity constitutes a key component of apathy (36). There is debate on the conceptual stability of behavioral symptoms of apathy, which could help explain our results. For example, Levy and Dubois (17) suggest a replacement of the term behavioral apathy with the concept of an “auto-activation deficit,” with reference to difficulties in activating thoughts or initiating motor programs necessary to complete an activity. Taken in this context, the IGT would not be a conceptually or a theoretically sound correlate of this dimension of apathy. Furthermore, the somatic marker hypothesis (33) that derives from research on performance on the task suggests that efficient performance requires the engagement of affective pre-biasing and tagging signals that aid the selection of adaptive behavioral choices (33). It is probably more reasonable to expect a relationship between impaired IGT performance and affective apathy symptoms. In line with this argument, Levy and Dubois (17) suggest that affective apathy may reflect an inability to associate those pre-biasing affective signals with ongoing and forthcoming behaviors.

## Conclusion

Results from this study suggest that the three domains of apathy might have distinct neurocognitive correlates whose association with apathy may get masked by treating apathy as a unitary syndrome. Fractionating apathy symptoms into affective, cognitive, and behavioral sub-dimensions may be a useful approach to understand apathy and may also have greater utility in research and clinical practice. This approach also takes into account the fact that processes that direct and sustain human motivated behavior are broad; ranging from those that process rewards and punishments, those that are engaged in planning and executive control, to those that initiate motor sequences or sequences of thought. Deficits in any of these processes can disrupt goal directed behavior and manifest as an apathetic state. As noted by Levy and Czernecki (2), the physiopathology of apathy may depend on which specific process is disrupted during the execution of goal directed behavior.

Several limitations of this study should, however, be mentioned. First, the sample of patients was small, and this limited the power of the study. A bigger sample size is needed to strengthen the relationships between the study variables, especially where the results neared significance. For this reason, the near significant results can only be interpreted with caution. Furthermore, the multiple comparisons made on the same sets of data increased the chances of making family-wise type 1 errors. In some cases, the theoretical foundation for picking up predictors for the hierarchical regression models was not robust enough. This is particularly the case in relation to the association of behavioral apathy symptoms with IGT performance. There is still a lot of gray areas around the conceptualization and definition of apathy, which makes it difficult to come up with a solid theoretical framework for research. Future studies may also investigate symptom–lesion relationships or use brain imaging techniques to investigate the neural substrates of the individual sub-domains of apathy. The study focus can also be sharpened a bit further. For instance, future studies may investigate how these sub-domains of apathy relate to deficits in recognizing specific emotions, or relate to specific sub-domains of depressive symptoms.

## Author Contributions

Progress Njomboro was involved in the conception and design of the study, collected and analyzed the data, and also did the write-up. Shoumitro Deb was involved in the conception and design of the study.

## Conflict of Interest Statement

The authors declare that the research was conducted in the absence of any commercial or financial relationships that could be construed as a potential conflict of interest.
